# Narrative update of clinical trials with antihypertensive drugs in children and adolescents

**DOI:** 10.3389/fcvm.2022.1042190

**Published:** 2022-11-21

**Authors:** Josep Redon, Tomas Seeman, Dénes Pall, Lagle Suurorg, Konstantinos Kamperis, Serap Erdine, Elke Wühl, Giuseppe Mancia

**Affiliations:** ^1^INCLIVA Research Institute, CIBERObn Institute of Health Charles III, University of Valencia, Madrid, Spain; ^2^Department of Pediatrics, 2nd Faculty of Medicine, Charles University Prague, Prague, Czechia; ^3^Department of Medical Clinical Pharmacology, University of Debrecen, Debrecen, Hungary; ^4^Tallinn Children's Hospital, Tallinn, Estonia; ^5^Department of Paediatrics and Adolescent Medicine, Aarhus University Hospital, Aarhus, Denmark; ^6^Hypertension and Atherosclerosis Center, Marmara University School of Medicine, Istanbul, Turkey; ^7^Division of Pediatric Nephrology, Center for Pediatrics and Adolescent Medicine, Heidelberg University Hospital, Heidelberg, Germany; ^8^University of Milano-Bicocca, Milan, Italy

**Keywords:** clinical trial (2.172), children, adolescents, antihypertensive drug, pharmacological treatment

## Abstract

**Introduction:**

To date, our knowledge on antihypertensive pharmacological treatment in children and adolescents is still limited because there are few randomized clinical trials (CTs), hampering appropriate management. The objective was to perform a narrative review of the most relevant aspects of clinical trials carried out in primary and secondary hypertension.

**Methods:**

Studies published in PubMed with the following descriptors: clinical trial, antihypertensive drug, children, adolescents were selected. A previous Cochrane review of 21 randomized CTs pointed out the difficulty that statistical analysis could not assess heterogeneity because there were not enough data. A more recent meta-analysis, that applied more stringent inclusion criteria and selected 13 CTs, also concluded that heterogeneity, small sample size, and short follow-up time, as well as the absence of studies comparing drugs of different classes, limit the utility.

**Results:**

In the presented narrative review, including 30 studies, there is a paucity of CTs focusing only on children with primary or secondary, mainly renoparenchymal, hypertension. In trials on angiotensin converting enzyme inhibitors (ACEI), angiotensin receptor blockers (ARBs), calcium channel blockers (CCBs) and diuretics, a significant reduction of both SBP and DBP in mixed cohorts of children with primary and secondary hypertension was achieved. However, few studies assessed the effect of antihypertensive drugs on hypertensive organ damage.

**Conclusions:**

Given the increasing prevalence and undertreatment of hypertension in this age group, innovative solutions including new design, such as ‘*n-of-1*', and optimizing the use of digital health technologies could provide more precise and faster information about the efficacy of each antihypertensive drug class and the potential benefits according to patient characteristics.

## Introduction

Globally, and particularly in developing countries, hypertension (HTN) is the most common disease of adulthood (1) and low rates of antihypertensive treatment and blood pressure (BP) control are the most important cause of the high cardiovascular morbidity and mortality worldwide (2). Even though its prevalence is much lower in children and adolescents than in adults, HTN has a great clinical importance also at a young age because BP elevation in young people makes the development of sustained HTN in adulthood more likely (3). Furthermore, in recent decades the number of young patients with a diagnosis of hypertension has been found to increase. This is in part because of the wider use of BP measurements (4) but unquestionably also to the increase of overweight and obesity in younger populations (5). Because HTN in adulthood has its roots in childhood, it is important to measure BP appropriately and diagnose pediatric HTN in a timely manner (6). Diagnostic criteria for elevated BP in children and adolescents are based on the concept that BP increases with age and body size, making it impossible to utilize a single BP level to define HTN, as done in adults. Hypertension is defined as systolic and/or diastolic BP persistently ≥95th percentile of the normative BP distribution, adjusted by age, sex and height measured on at least three separate occasions. Consistent with the physiological body growth adult cut-points 140/90 mmHg are applied for adolescents 16 years and older (Table 1) (4).

**Table 1:** Criteria for the methods to establish dosing recommendation and safety of antihypertensives from EMA (15).

**Criteria of efficacy**	
**Blood pressure values**	
Reduction of BP	**X**
Absolute or percent change in systolic or diastolic blood pressure	**X**
Trial design A/B–change from baseline to the end of the treatment period + inter-dosing interval (trough)	
Trial design C/D–change in blood pressure from the last on-treatment visit to the end of withdrawal period	
**Morbidity and mortality**
Establishing an effect on morbidity and mortality endpoints is not required in pediatric licensing trials of antihypertensive medicinal products	**X**
Post-authorization long-term follow up and observational research are encouraged	**X**
**End organ damage**
Albuminuria	**X**
Left ventricular hypertrophy and/or dilatation	**X**
Assessment of presence and progression of other types of organ damage is advisable in longer-term studies	**X**
**Methods to assess efficacy**	
**Reduction in blood pressure values**	
Office BP systolic or diastolic	**X**
Home BP and ABPM is encouraged	**X**
**Changes in end organ damage**	
Kidney: GFR and albuminuria/proteinuria	**X**
Left ventricular mass or dilatation by height	**X**
Arterial wall (thickening in the intima-media complex)	**X**
**Patients**
Hypertensive diagnosed	**X**
Youngest age groups after the safety have been established in the older patients, especially in studies involving infants < 6 months	**X**
Differentiated between essential and secondary forms of HTN	**X**
Unnecessary studies in children should be avoided. This is not the case for products with new mechanism of action and in younger age groups where dedicated dose-ranging and safety studies are always necessary.	**X**
The use of placebo or fixed low dose of the product require ethical acceptability and safety aspects when evaluating the feasibility of studies in the most severe forms of HTN	**X**
Stratification of randomization according to the etiology or patient characteristics needs to be discussed when has been identified as potentially useful	**X**
**Design**
**Pharmacology studies**
PK data for all relevant pediatric age groups should be provided	**X**
Bioavailability half-life, C_max_ and T_max_ in the various age groups and for parent and metabolites	
A reasonably precise estimate of which range of doses provides sufficient exposure, equivalent to the doses determined to be efficacious in adults with hypertension, is needed.	**X**
PD considerations to be addressed by the applicant include, but are not limited to, possible differences in pharmacology, metabolism and PK/PD relationship/dose-response slope according to age	**X**
For children 1 to < 6 years of age, a formulation that allows adequate dosing flexibility is a must to assure reliable administration and accurate weight- adjusted dosing	**X**
**Therapeutic studies**
The main aim of the pediatric development is to establish the therapeutic dose as well as tolerability, palatability (where appropriate), short- and long- term safety.	**X**
Double blind randomized study design with or without a placebo arm (no in youngest, < 6 years) and more severe HTN	**X**
Rescues treatments in case of insufficient response should be predefined	**X**
Dose ranges enough wide	**X**
Dose ranges will also depend on age-specific differences suggested by PBPK-modeling and/or pediatric PK data	**X**
Doses providing exposure from slightly lower than the lowest approved adult dose up to somewhat higher than the highest approved dose in adults (unless restricted by safety concerns) could be considered	**X**
**Safety**
Short-term tolerability and safety data should be collected in the controlled studies and compared with the known safety profile in adults.	**X**
The trial program is expected to have a total of no < 300 pediatric patients for safety reasons to identify adverse reactions occurring with a 1% frequency.	**X**
Extension studies with individual dose titration after completion of the short-term studies or dedicated safety studies are needed for collection of longer-term safety data.	**X**
At least 12 month extension studies are necessary to allow investigation of long-term safety in terms of growth (head circumference, weight and height) and development, including neurocognitive development	**X**
Younger age groups (infants, children under 6 years of age) have to be adequately represented and may need to be followed up longer (e.g., 24 months).	**X**
Identified safety concerns from adult or non-clinical studies may necessitate further data collection,	**X**

PK, pharmacokinetics; Cmax, highest concentration; Tmax, time it takes for a drug to reach the maximum concentration; PD, pharmacodynamics.

Currently primary HTN is the most frequent cause of high BP in children and adolescents with a close association with overweight and obesity (7). As in adulthood, the first therapeutic step to adopt under these circumstances should be non-pharmacological treatment, i.e., modifications of incorrect lifestyles that may contribute to BP elevation (8, 9). However, in children where such a strategy fails, pharmacological treatment is indicated (4) and in young people with symptomatic HTN, secondary HTN, target organ damage, chronic kidney disease or diabetes mellitus, pharmacological treatment should be considered as first line therapy. Unfortunately, however, knowledge of what should be the optimal first step drug or drugs in children and adolescents is much more limited than in adults (4, 8). In addition, no or few good-quality long-term outcome data are available to guide pediatricians in selecting medication to treat HTN, which means, that treatment is often based on experience rather than on evidence. In the absence of evidence, use of “off-label” drugs is also common (10), further complicating the appropriate management of pediatric HTN and making it a challenging task for pediatricians. Many of whom feel uncomfortable treating a hypertensive child, also because recognition of a HTN state is more difficult than in adults. Nevertheless, during the last decades, childhood HTN has been studied more rigorously, to optimize BP measurements, collect normative data and establish diagnostic work-up guidelines. To-date the development of worldwide adopted recommendations has improved our ability to diagnose pediatric HTN to an extent superior to that of HTN management, which has made much less progress (4).

Clinical trials of antihypertensive drugs in the adult population have yielded in-depth information about their pharmacokinetics and pharmacodynamics, including BP lowering efficacy, effects on hypertension-related outcomes and safety for all major classes of antihypertensive medication. Data on optimal drug doses, best combinations, and differences in efficacy among the different drug classes have also been obtained. In contrast, in the pediatric population, paucity of studies is the rule, which is a major shortcoming because what works in adults does not necessarily work in children and adolescents. Furthermore, most drug formulations are not adapted for use in the pediatric age.

**Figure 1:**
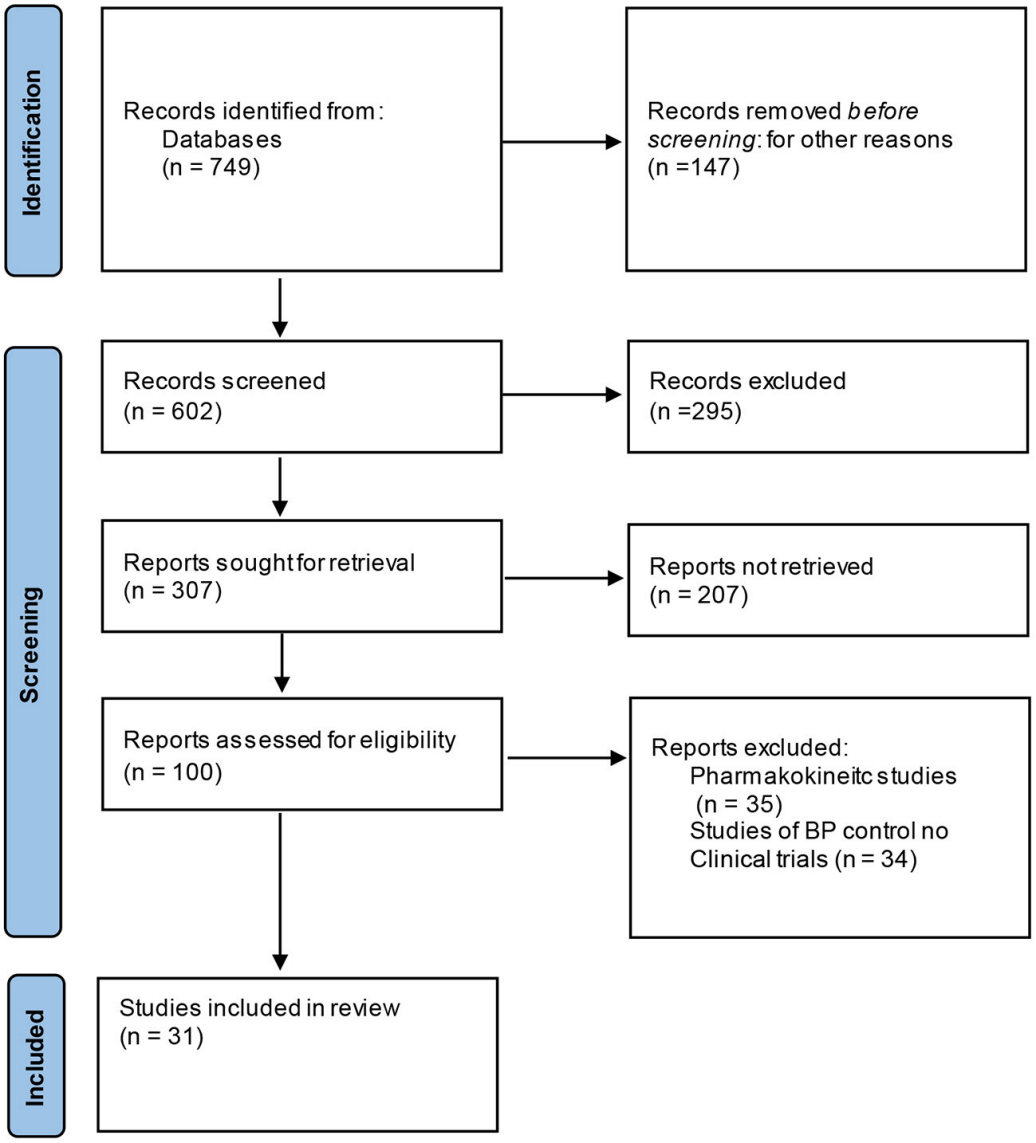
Flow-chart of the studies selected.

The present review focuses on CTs of antihypertensive drugs in primary and secondary HTN of children and adolescents, with emphasis on future research needed in this age population. PRISMA system have been used (11) to select the studies to be included with descriptors: clinical trials, antihypertensive drugs and children and adolescents, in PubMed. The flow diagram is in Figure 1.

## Regulatory agencies and hypertension drug treatment

During the last three decades, Regulatory Agencies have effectively acted to provide better information about the use of drugs for pediatric treatment and to promote their availability. In the US, incentives were first authorized by the Food and Drug Modernization Act of 1997 such as the 6 month prolongation of the market patent for drugs which were tested by clinical trials in children, as well as the possibility to perform clinical trials with off-patent drugs. This was reauthorized in 2002 by the *Best Pharmaceuticals for Children Act (BPCA)* (12), and permanently reauthorized by FDA in 2012 under the *FDA Safety and Innovation Act* (13).

Similar actions were taken in Europe by the *Regulation of Medical Products for Pediatric Use* (14). The *Pediatric Committee of the European Medicines Agency (PDCO)* is the scientific committee responsible for activities connected with medicines to be used in pediatrics and for their development in the European Union *via* scientific support and help to data analysis in the area of pediatrics. The PDCO was created by the pediatric regulations that came into force in 2007, with the aim of improving the health of the European Union's pediatric population *via* development and increasing the availability of *ad hoc* medicines. A *Pediatric Investigation Plan (PIP)* promoting research activities has also been launched, including PIPs for treatment of cardiovascular disease, HTN in particular (15), although at present the numbers of PIPs in this area lags behind other therapeutic areas.

As shown in Tables 1, 2 the two Regulatory Agencies have also established the requirements for the approval and conduction of CTs. Although some requirements are in agreement, others differ between the two Agencies (16, 17), with the main differences involving methods of measuring BP, assessment of organ damage, range of drug dose to be used, and time for extended observation after completion of a trial.

**Table 2:** Criteria for the methods to establish dosing recommendation and safety of antihypertensives from FDA agencies (16).

**Criteria of efficacy**	
**Blood pressure values**	
**Reduction of BP**	
Absolute or percent change in systolic or diastolic blood pressure	**X**
Trial design A/B–hange from baseline to the end of the treatment period + inter-dosing interval (trough)	
Trial design C/D–change in blood pressure from the last on-treatment visit to the end of withdrawal period	
**Morbidity and mortality**	
Post-authorization long-term follow up and observational research are encouraged	**X**
**Methods to assess efficacy**	
**Patients**	
Hypertensive diagnosed	**X**
>90^th^ percentile if concurrent conditions present	**X**
Demographic criteria: >50% pre-adolescents subjects; 40–60 black subjects, both sexes	**X**
**Design**	
**Pharmacology studies**	
PK data for all relevant pediatric age groups should be provided	**X**
Bioavailability half-life, C_max_ and T_max_ in the various age groups and for parent and metabolites	**X**
Blood levels should range from less than those achieved with the lowest approved adult dose to more than those achieved with the highest generally used adult dose	**X**
For children 1 to < 6 years of age, a formulation that allows adequate dosing flexibility is a must to assure reliable administration and accurate weight- adjusted dosing	**X**
**Therapeutic studies**	
Trial duration typically 2 weeks but longer if a period of dose titration is needed	**X**
For statistical consideration ≥80% power to detect a 3 mmHg change in blood pressure of conventional (p < 0.05, two-sided) statistical significance	**X**
**Safety**	
At least 12-month extension studies are necessary to allow investigation of long-term safety in terms of growth (head circumference, weight, and height) and development, including neurocognitive development	**X**
Specific safety concerns during the studies in infants may need to be addressed by stepwise recruitment to the trials (interim safety data analysis before the inclusion of the youngest patients)	**X**
Trials should include a 1 year open-label treatment period to evaluate adverse events, growth (change in head circumference, weight, length, or height) and development (milestones, school performance, neurocognitive testing)	**X**

PK, pharmacokinetics; Cmax, highest concentration; Tmax, time it takes for a drug to reach the maximum concentration; PD, pharmacodynamics.

## Clinical trial challenges

### Barriers

Research on children in CTs faces ethical, epidemiological, and economic difficulties or barriers, which have some special characteristics in the case of antihypertensive drugs. Research failures may occur because the results do not reach statistical significance and thus the efficacy of a tested drug cannot be proven. Other reasons are inconsistent results, controversial results or project failure because the budget has been overspent, the project targets have not been achieved or deadlines haven't been met (18).

In a review of CTs on failure rates and causes of the use of drugs for hypertension care in children and adolescents since 2000 (search keywords “pediatric drug therapy,” “hypertension,” “clinical trials” and “fail of trials”) nine of the sixteen pediatric antihypertensive drug trials failed to show a dose response (19) due to unskilled project manager, unproductive team, complexity of protocol, the dilemma of “*Project Completion Targets” vs. “Eligibility of the Volunteers*,” poor training and poor verification, ethical issues and data quality (20, 21).

Finally, early discontinuation and lack of publication of study findings are common in registered pediatric CTs. Targeted efforts are needed to support trial completion and timely result dissemination to strengthen evidence-based pediatric medicine (22).

### Previous analysis assessing BP lowering effect and safety

As in adults, in children and adolescents the BP-lowering effect of drug treatment may be influenced by several factors, including age, sex, weight, and severity of baseline hypertension, which makes achievement of conclusive information on between-drug differences in efficacy far from simple.

In a Cochrane review article (23) including 21 randomized CTs, a total of 3,454 hypertensive children and adolescents were enrolled when at least a 2 week comparison was made between (a) monotherapy or combination therapy with either placebo or another medication or (b) different doses of the same drug. Despite use of random effect models the authors emphasized that safe conclusions could not be made due to lack of sufficient data. Nevertheless, they stated that the agents tested, i.e., ACEIs, ARBs and CCBs did not exhibit a consistent dose-response relationship, although all of them appeared to be safe, at least within the short-term context of the studies.

A more recent meta-analysis (24) tried to assess more uniform and higher clinical quality CTs by selecting 13 trials with a randomized placebo-controlled design, more than 50 patients enrolled, and a follow-up of at least 4 weeks. Patients affected by secondary forms of hypertension, which may benefit from specific and targeted therapies, were not systematically excluded. The results were rather inconclusive because, despite the more demanding selection of the studies, the results remined heterogeneous and the follow-up time short. The authors highlighted that the observations nevertheless increased the available experience with drugs that block the renin angiotensin system (i.e., ACEIs and ARBs), because these drugs accounted for the greater proportion of treatment in the patients recruited.

### Update of present knowledge

A total of 31 (25–55) CTs have been summarized in the present review, the majority (*n* = 20), (26, 29–38, 40, 41, 45, 47–53) (Table 3) including children with both primary and secondary HTN. Fewer CTs investigated antihypertensive drugs focusing only on children with primary HTN and taking into account concomitant obesity (48) or race (46). The majority children participating in CTs on secondary HTN had renal disease as the first cause of HTN (27, 31, 34–40, 42, 43, 47, 49–55). Some of the CTs in renal disease assessed changes in albuminuria or proteinuria (35, 36, 52, 53).

**Table 3:** Main characteristics of clinical trials in blood pressure reduction.

**Drug/s** **Reference (__)**	**Age range (Sample size)**	Low dose^*^(daily)	**Maximal dose (daily)**	**Dosing interval**	**Baseline BP SBP/DBP**	BP (mmHg)^**^ SBP^***^ **DBP**	**Population additional comments**
**Diuretics**							
HCTZ vs. Clonidine (Cl) Falkner et al. (25)	13–19 (28)	25 mg/day vs. 0.1 mg/kg/day	50 mg/day vs. 0.2 mg/kg/day	oid	Hczt 146/96 Cl 145/97	Hctz 10 Cl 10-10^***^ Hctz 4 Cl 7-7	
Chlortalidone vs. Propanolol Bachman (26)	(9)	0.3 mg/kg	2 mg/kg up to 50 mg	oid		15 to 22,3	
Eplerenone vs. placebo Li et al. (27)	4–16 (304)	25 mg/day	50 mg/bid	oid/bid	128/70	7.6/7.9^***^ 2.7/2.8	Primary + secondary
**Beta-blockers**							
Bisoprolol/HCTZ vs. placebo Sorof et al. (28)	6–17 (94)	2.5 mg/day 6.25 mg/day	10 mg/day 6.25 mg/day	oid	133/82	4.9/9.3^***^ 2.9/7.2	
Metoprolol vs. placebo Batisky et al. (29)	6–17 (138)	0.2 mg/kg	2 mg/kg	oid	128/95	7.7^***^ 4.9	
Esmolol Tabbutt et al. (30)	1–6 (116)	0.1 mg/kg iv	5 mg/kgv iv	Ibolus and iv infusion	No reported∧	9.6^***^ –	After repair aortic coarctation
Atenolol vs. Enalapril Di Salvo et al. (31)	6–20 (49)					A 9.0 E 8.0^***^	After repair aortic coarctation
**Calcium channel blockers**							
Amlodipin, Nifedipin, Felodipin Rogan et al. (32)	9–17 (9)	0.1 mg/kg	5 mg	oid	No reported∧	No reported	Renal transplants No differences between the drugs
Felodipine vs. placebo Tractman et al. (33)	6–12 (128)	2.5 mg/day	10 mg/day	oid	No reported∧	0.1^***^ 4.9	
Amlodipine vs. placebo Flynn et al. (34)	6–16 (352)	2.5 mg/day	5 mg/day	oid	137/74	7.3/9.1^***^ 3.7/4.4	Primary + secondary
**Angiotensin converning enzyme inhibitors**							
Ramipril vs. placebo Soergel et al. (35)	5–18 (12)	1.5 mg/m^2^	10 mg	oid	No reported∧	5 (24 h)^***^ –	Only renal disease
Ramipril vs. placebo Seeman et al. (36)	2–19 (29)	1.5 mg/m^2^	10 mg	oid	No reported∧	11 (day) 8 (night)^***^ –	Only renal disease
Ramipril Wühl et al. (37)	3–8 (385)	6 mg/m^2^	10 mg	oid	Mean BP 89.5 (24 h)	Mean BP 11.6 (24 h)	Renal disease hypertensive and normotensive
Enalapril vs. placebo Wells et al. (38)	6–16 (110)	0.625 to 5 mg/day	20–40 mg	oid	129/86	6.8/11.0^***^ 7.1/10.2	>50% renal disease
Lisinopril Soffer et al. (39)	6–16 (115)	2.5–5	50–100 mg	oid	129/90	5.0/15.0^***^ 7/16	>50% renal disease
Fosinopril vs. placebo Li et al. (40)	6–16 (255)	0.1 mg/kg/day	6 mg/kg/day	oid	134/71	11/11^***^ 4.5/5.1	21% renal disease
Enalapril (E) vs. Valsartan (V) Schaffer et al. (41)	6–16 (281)	0.1 mg/kg/day	6 mg/kg/day	oid	134/79 133/78	E 11 V 11^***^ E 4.5 V 5.1	
**Angiotensin-AT1-receptor blockers**							
Losartan vs. placebo Shahinfar et al. (42)	6–16 (175)	2.5–5	50–100 mg	oid	129/89	4.4/10.0^***^ 6.0/12.2	>50% renal disease
Valsartan vs. placebo Flynn et al. (43)	1–5 (90)	5 mg/day	89 mg/day	oid	118/71	8.5^***^ 5.7	63% renal disease
Candesartan vs. placebo Trachtman et al. (44)	6–17 (233)	2–4 mg/day	16–32 mg/day	oid	133/78	4.9-7.5^***^ 3.0/6.2	
Telmisartan vs. placebo Wells et al. (45)	6–18 (77)	1 mg/kg/day	2 mg/kg/day	oid	131/79	9.0/14.0^***^	
Olmesartan vs. placebo Hazan et al. (46)	6–16 (302)	2.5–5 mg/day	20–40 mg/day	oid	130/78	7.8/12.6^***^ 5.5/9.5	Lower response in black
Candesartan Schaefer et al. (47)	1–5 (93)	0.05 mg/kg	0.4 mg/kg	oid	112/70	12^***^ 11	80% renal disease
Valsartan vs. placebo Meyers et al. (48)	6–16 (261)	10–20/day	80–160 mg/day	oid	No reported∧	7.0/13.0^***^ 4.0/9.0	Obese and non-obese
Valsartan vs. placebo Wells et al. (49)	6–16 (261)	10–20/day	80–160 mg/day	oid	**133/77**	7.9/11.5^***^ 4.6/7.4	18% renal disease
Losartan vs. Amlodipino Webb et al. (50)	1–17 (28)	L0.7 mg/kg A0.1 mg/kg	L1.4 mg/kg A1 mg/kg	oid	No reported∧	1.9^***^ +3.9	Alport syndrome Hypertensive and normotensive No significant BP reduction
Valsartan vs. placebo Schaefer et al. (51)	1–5 (75)	0.25 mg/kg	4 mg/kg	oid	No reported^∧^	8.3/14.4^***^ –	61% renal disease
Losartan Webb et al. (52)	6 mo−6 (101)	0.1 mg/kg	100 mg	oid	111/69	7.9^***^ –	66% renal disease
Valsartan open label Lou-Meda et al. (53)	6–17 (150)	40 mg/day	160–320 mg/day	oid	135/82	11.0/19.0^***^ 9.6/12.0	With and without CKD
Valsartan vs. placebo Jankauskiene et al. (54)	1–5 (127)	0.25 mg/kg	4 mg/kg	oid	No reported^∧^	4.4^***^ –	53% renal disease
Azilsartan open label Ito et al. (55)	6–15 (55)	2.5–5 mg/day	20–40 mg/day	oid	123/72 136/71	8.8/15.4^***^ 10.3/13.6	Secondary HTN

+oid, once a day; bid, twice a day.

^*^Body weight dependent.

^**^Lowest to highest placebo subtracted.

^***^Indicated separation between reductions in SBP (upper) or in DBP (down). ^∧^No reported the baseline values was in some studies no presented and only reduction of BP values are reported. In other studies, the baseline was reported for different categories of body weight and the lowest to highest reduction were reported. Gray shaded lines: studies with secondary hypertension.

Among the CTs specifically addressing primary HTN, one study analyzed the antihypertensive efficacy of Valsartan (48) in obese and non-obese hypertensives, and found that BP reduction was similar in the two groups. In another specific CT on primary HTN, Olmesartan, an angiotensin receptor blocker, was less effective in reducing BP in African American children compared to Caucasians (46), a result in line with data available in the adult population.

Regarding secondary vs. primary HTN, most CTs did not perform a separate analysis of the BP lowering effect of study medication in children with primary and secondary HTN.

In the mixed cohorts, in which ACEI, ARBs or diuretics were used a significant reduction of both systolic (SBP) and diastolic BP (DBP) was observed. This was the case also in the only study in which amlodipine was used. In this study however, a separate analysis of children with primary and secondary HTN was made. The results showed that there was no effect of the underlying cause of HTN on BP response (34). Thus, the authors concluded that, at least with regard to amlodipine, the BP lowering effect of drug treatment in children with secondary HTN is not different from that in children with primary HTN. Taken together, the data suggest that in children with primary vs. secondary HTN there may be no significantly different BP lowering effect of a variety of antihypertensive drugs, a conclusion supported by data in adults.

Four studies analyzed the impact of antihypertensive drug treatment only in secondary HTN. In renal posttransplant patients one study compared three CCBs, i.e., amlodipine, nifedipine and felodipine, and found no difference in the BP-lowering effect among them (32). In the second study losartan and amlodipine both resulted in a significant decrease of SBP but not of diastolic BP compared to placebo in children with Alport syndrome (51). In the last two studies, esmolol and atenolol, beta-blockers, effectively reduced BP in the post-operative phase of surgery for aortic coarctation (30, 31).

From the above review it is clear that data on antihypertensive drugs in the young age are scarce. Beside the limited information on the BP lowering effect of different drugs and the probable similarity of antihypertensive drug treatment effects in primary and secondary HTN, no adequate data are available on the effect of different timing of drug administration, the relationship with food intake, the effect on BP reduction during sleep and the comparison of different agents within the same drug class. Data about the effect of antihypertensive drugs on hypertensive target organ damage are also very limited and the safety profile of the antihypertensive drug administration, although addressed by some CTs, almost entirely lacks of longer-term information as well as of information in children with other health problems such as lung disease, cardiac disease, and others.

Overall, the available evidence appears to allow a relatively safe choice of at least the class of antihypertensive drugs in children with secondary HTN. Most other clinical considerations, however, are still largely depending on the underlying pathophysiology and the presence of concurrent disorders such as diabetes mellitus, chronic kidney disease, proteinuria, overweight and more.

The studies available in the ClinicalTrials.gov Search Results (56) (consulted 07/05/2022) include only six studies in different stages of conduction but not published yet (Table 4). The design does not seem to cover the previously mentioned issues.

**Table 4:** ClinicalTrials.gov search results 07/05/2022 (56).

A study of the effectiveness and safety of ramipril in the treatment of hypertension in children and adolescents	Terminated	Has results	HTN	Ramipril Placebo
Evaluation of the safety, efficacy and pharmacokinetics of MICARDIS^®^ (Telmisartan) in children and adolescents with hypertension	Completed	No results available	HTN	Telmisartan Placebo
Assessment of efficacy and safety of olmesartan medoxomil in children and adolescent patients with high blood pressure	Completed	Has results	HTN	Olmesartan medoximil Placebo
Safety study of lisinopril in children and adolescents with a kidney transplant	Completed	Has results	HTN	Lisinopril
Treatment of pediatric hypertension with altace trial	Completed	No results available	HTN	Ramipril
A study of valsartan used to treat hypertension for up to 13 months in hypertensive children ages 6–16 years of age	Completed	No results available	HTN	Valsartan

### *n-of-1* trials

Given the limitations of the CTs performed in the last 20 years, new research approaches are needed to provide evidence on how to select appropriate antihypertensive medications in children, in terms of efficacy and tolerance as well as persistence of the effect over prolonged time periods.

The ***n-of-1 trial*** (a.k.a. single-patient trials) is a promising approach to identify the most successful treatment for diseases that require treatment during prolonged periods of time. Based on a document released in 2014 by the Agency for the Health Care Research and Qualities (57), *n-of-1* trials, is a form of prospective research in which different treatments are evaluated in an individual patient over time.

The approach has been applied to HTN using ambulatory blood pressure monitoring (ABPM). In a randomized trial (58), three drugs from different classes were selected and started in patients in whom HTN had been confirmed by ABPM. The first drug was given during the first 2 weeks, the second drug during weeks 3 and 4 and the third drug during weeks 5 and 6. At the end of each 2 week period a 24 h ABPM was performed. Once the first circle (6 weeks) was finished, the drug with unacceptable side effect profile or minimal BP reduction was discarded and the procedure was repeated for the remaining two drugs. In the end the drug with the best treatment adherence, patient satisfaction, and BP control was selected. It should be noted that the above design does not meet with universal agreement because compliance can be challenging for both patients and physicians, although the results can be useful in patients who require long-term BP control.

## Future perspectives

Innovative solutions are needed to optimize the traditional testing of drugs. The application of rapidly evolving digital health technologies and artificial intelligence in HTN healthcare and research (digital hypertension) holds promise to provide further insights into the understanding of pathophysiology as well as the identification of therapeutic targets and efficacy of antihypertensive drugs.

The stringent isolation measures adopted during the pandemic have strongly promoted telemedicine practices that provide information *via* communication technologies that use several distinct methods (59).

A prospective study evaluating 263 interviews between health care professionals and children with chronic diseases suggests that telemedicine applications are useful tools not only during pandemics but also in daily practice (60). One application now frequently used in managing HTN is BP telemonitoring (BPT) (61). Although patients' compliance might be a potential limitation, a systematic review article points out that all current studies regarding the efficacy of BPT exhibit several benefits for long-term follow-ups, including reduction of health care costs and improvement of outcomes in the pediatric population (62). This opens also opportunities to improve drugs.

Artificial intelligence (AI) is another promising tool for the management of patients with high blood pressure and can also improve the assessment of drug efficacy (63). Machine learning methods differ significantly from traditional statistical methods. While conventional statistics focuses mainly on the conclusions, AI-derived statistics generally concentrates on prediction and decision-making. Therefore, they are commonly used as risk-stratifying and scoring tools (64, 65). However, the role of AI techniques in the management of HTN remains unclear and controversial due to several limitations, such as requiring large amounts of data, lack of data quality, lack of standardized models that can be reliably used for different populations, dependence mostly on laboratory findings without adequate environmental factor assessments, necessity to retrain the neural network whenever a significant change is made in the target population, and insufficient training of clinicians in bioinformatics and data science (63, 66, 67).

## Conclusions

Despite the traditional belief that HTN is a rare condition in children, there is accumulating evidence that elevated BP is increasingly common in both children and adolescence. Despite the abundance of different pharmacological agents designed to treat HTN these are mostly studied in adults and only over the last years industry and authorities have identified the need of well conducted randomized trials of pharmacological treatment in childhood HTN.

Legislation changes have pushed for pediatric studies, but we are still far from establishing a confident level of knowledge in HTN management for children. CTs available today lack hard evidence to recommend any class of antihypertensive medication over the others as first line in children. Furthermore, the impact of pharmacological therapy on cognitive development and growth is not sufficiently studied.

Overall, it is beyond any doubt that we lack important knowledge when it comes to pharmacological antihypertensive treatment in children. The increasing number of children to be treated rises the need for large multicenter randomized trials to investigate the best treatment strategies for each child, to identify optimal dosage regimes and improve the long-term safety of antihypertensive medication in children. In addition to classical CTs, new approaches will contribute to get more grounded information.

## Data availability statement

The original contributions presented in the study are included in the article/supplementary material, further inquiries can be directed to the corresponding author.

## Author contributions

JR, GM, and SE contributed to conception and design of the study. JR wrote the first draft of the manuscript. JR, SE, EW, GM, DP, TS, LS, and KK wrote sections of the manuscript. All authors contributed to manuscript revision, read, and approved the submitted version.

## Funding

This article is based upon work from COST Action HyperChildNET (CA19115), supported by COST (European Cooperation in Science and Technology).

## Conflict of interest

The authors declare that the research was conducted in the absence of any commercial or financial relationships that could be construed as a potential conflict of interest.

## Publisher's note

All claims expressed in this article are solely those of the authors and do not necessarily represent those of their affiliated organizations, or those of the publisher, the editors and the reviewers. Any product that may be evaluated in this article, or claim that may be made by its manufacturer, is not guaranteed or endorsed by the publisher.
